# Composites of Poly(vinyl chloride) with Residual Hops after Supercritical Extraction in CO_2_

**DOI:** 10.3390/polym13162736

**Published:** 2021-08-15

**Authors:** Jacek Mirowski, Rafał Oliwa, Mariusz Oleksy, Edward Rój, Jolanta Tomaszewska, Kamila Mizera, Joanna Ryszkowska

**Affiliations:** 1 Faculty of Chemical Technology and Engineering, UTP University of Science and Technology, Al. Prof. S. Kaliskiego 7, 85-796 Bydgoszcz, Poland; jaca@utp.edu.pl (J.M.); jolat@utp.edu.pl (J.T.); 2 Department of Polymer Composites, Faculty of Chemistry, Rzeszow University of Technology, 35-959 Rzeszow, Poland; molek@prz.edu.pl; 3 Łukasiewicz Research Network–New Chemical Syntheses Institute, Al. Tysiąclecia Państwa Polskiego 13a, 24-110 Puławy, Poland; edward.roj@ins.lukasiewicz.gov.pl; 4 Faculty of Materials Science and Engineering, Warsaw University of Technology, Wołoska 141, 02-507 Warsaw, Poland; Kamila.Mizera.dokt@pw.edu.pl (K.M.); joanna.ryszkowska@pw.edu.pl (J.R.)

**Keywords:** PVC, residual hops, polymer composites, natural particles, thermal stability, flame resistance, mechanical properties

## Abstract

The common applications of poly(vinyl chloride) (PVC) in many industries mean that the topic of recycling and disposal of post-consumer waste is still very important. One of the methods of reducing the negative impact of PVC waste on the natural environment is to use technological or post-consumer waste of this polymer to produce new composite materials with favorable utility properties, with the addition of natural fillers, among which agro-waste, including hop residue, is deserving of special attention. In this study, the effect of the addition of residual hops (H) on the mechanical and physicochemical properties of poly(vinyl chloride) was investigated. PVC blends containing 10, 20 and 30 wt % of hop residue were mixed in an extruder, while the specimens were obtained by the injection molding method. It was observed that the addition of H increased their thermostability, as shown by a Congo red test. Furthermore, thermogravimetric analysis showed that the degradation rate of PVC/H composites in the first and second stages of decomposition was lower in comparison with unmodified PVC. In turn, composite density, impact strength and tensile strength decreased significantly with an increasing concentration of filler in the PVC matrix. At the same time, their Young’s modulus, flexural modulus and Rockwell hardness increased. Flame resistance tests showed that with an increasing residual hop content, the limiting oxygen index (LOI) decreased by 9.0; 11.8 and 13.6%, respectively, compared to unfilled PVC (LOI = 37.4%). In addition, the maximum heat release rate (pHRR) decreased with an increasing filler content by about 16, 24 and 31%, respectively. Overall, these composites were characterized by a good burning resistance and had a flammability rating of V0 according to the UL94 test.

## 1. Introduction

In 2018, approximately 44.3 Mt of poly(vinyl chloride) (PVC) was produced worldwide [1]. The high interest in PVC production is due to its durability, light weight, strength, fire resistance, insulating properties and low permeability [2]. It is used in the manufacture of prefabricated sandwich structures and finished products such as window frames, profiles, floors, wall coverings, pipes, cable insulation, garden hoses, inflatable pools and geomembranes [2,3,4,5,6]. PVC comes in two basic forms: rigid (sometimes abbreviated as RPVC or UPVC) and flexible [2].

The widespread use of durable PVC, however, generates a large amount of waste, and a great challenge for scientists is developing processes that reduce the impact of PVC post-consumer waste on the environment. Undoubtedly, reducing waste is possible through recycling, and among the many methods, mechanical recycling is the most effective and environmentally friendly: grinding PVC for use as polymer filler [7,8], a polymer blend [9] or an aggregate substitute in concrete mix [10,11,12]. Another method is to add natural filler from the agro-food industry (agro-waste), thereby supporting biodegradability, renewability and the economy [13,14,15,16,17,18]. An advantage of these composites is that they can be manufactured using traditional plastic processing techniques, making it possible to form products of different shapes and sizes for use in various industries, such as construction and automotive and in consumer applications [13,19]. In addition, PVC-processed waste that is not recyclable can be used to produce composites similar to polymer–wood [20]. Another advantage of using natural fillers is the possibility of increasing the flame resistance of composites. Currently, bio-based flame retardants are becoming attractive and are the subject of many studies on new flame retardants, as they fulfil the green synthesis and non-toxicity requirements and have good efficiency [21]. Admittedly, rigid poly(vinyl chloride) is a material with a very high flame resistance, as confirmed by an oxygen index of up to 50% and a significantly lower heat release capacity compared to other flammable polymers [22]. However, the addition of modifiers, including plasticizers, to PVC results in a reduction in the chlorine content of the mass, leading to a significant deterioration in flame resistance, especially of flexible PVC [23]. Therefore, the current reports in the literature on bio-based FRs focus on the flame retardancy of flexible PVC by using nitrogen- or phosphorous-containing bio-plasticizer [24,25,26], phytic acid salts [27,28,29], lignins [30] and chitosan [31,32,33]. In the case of composites with a matrix of flammable polymers, also the addition of natural fillers such as kenaf [34], flax [35], bamboo [36], agave waste [37] and abaca [38] results in increased flame resistance. In contrast, there is currently a limited number of papers on the flame resistance of hard PVC matrix composites with the addition of natural fillers. Dutta and Kumar prepared composites containing a 40 part per hundred (phr) rise husk (RH) and 0–10 phr montmorillonite (MMT). The researchers observed that composites containing only RH had the lowest thermal stability and thermolysis residue, and an oxygen index of 39% [39]. In contrast, replacing the part of RH by the addition of 5 phr of microcrystalline cellulose (MCC) resulted in an oxygen index of 44.4%. However, the obtained value was still the lowest among all composites containing 35 phr RH, 0–5 phr MCC and 0–5 phr MMT, due to a poor dispersion and agglomerate formation [40].

One major source of agro-waste is beer production, which in 2019 amounted to approximately 1.91 billion hectoliters worldwide [41]. In recent years, valuable substances for the food, pharmaceutical and cosmetics industries have been extracted from the waste of hops, among other plants, using carbon dioxide under supercritical conditions [42]. Two parts of the plant can be subjected to this process: the first consists of leaves (tobacco and tea), flowers and stem fragments, the leaves and tops of fibrous hemp and hop cones; the second is seeds from flax, hemp, rapeseed, dill, coriander, black currants and carrots [42]. The great value of supercritical carbon dioxide extraction lies in the purity of the substances it produces: they contain no solvents and little moisture and, per unit weight, contain more lipids and protein than the raw material before extraction. Furthermore, agro-waste, after being extracted using supercritical CO_2_, has a lower density, higher porosity and a lower content of dye and other extracted chemical compounds.

After extraction, the remainder of the plant is burned as waste, but it could be used as filler in the production of PVC composites. Because Supercritical CO_2_-treated hop waste is sterile, it is suitable for long-term storage, and composites produced with it have excellent thermal, chemical and wear resistant quality; improved stiffness; water and oxygen impermeability and dimensional stability [3,43].

A continuous high demand for PVC in many industries industrial, technical and everyday applications means that the modification of this polymer with the use of natural fillers in order not only to improve its functional properties, but also to increase its biodegradability and recyclability and, thus, to reduce the negative impact on the natural environment, is an important aspect in the context of increasing its application possibilities. One of the groups in the natural environment is agro-waste. Apart from the fact that they are characterized by a low price, low density, processing and non-corroding ability, agro-grapevines are also used in polymer composites, which has a positive effect on reducing waste generated after food processing. Undoubtedly, hop residues, which are a source of lignocellulose, can also be an important natural filler used in polymer composites. Residual hops, such as other natural fillers, also contain a number of other ingredients such as proteins, waxes, fats, pectins, etc., which influence both the processing and final functional properties of PVC.

However, to the best of the authors’ knowledge, there is no information in the literature about using hop residue as a filler for poly(vinyl chloride) even though our previous work indicated the possibility of using agro-waste as a low-cost and eco-friendly filler for polymer plastics [17]. In addition, there is still insufficient information about flammability from PVC containing natural fillers, and available studies show that the addition of natural fillers may reduce flame resistance. Hence, the objective of this paper is to evaluate the possibility of manufacturing PVC composites with a hop residue filler obtained after being extracted using supercritical CO_2_. For this purpose, PVC composites containing 10–30 wt % of filler were prepared. The influence of the filler on the mechanical properties, flammability and structure of the composites was investigated. The results of the research will extend our understanding of the properties and possible applications of PVC composites containing agro-waste.

## 2. Materials and Methods

### 2.1. Materials

An unplasticized PVC S61 dry blend (Anwil S.A., Włocławek, Poland) with composition described in [17] was used as the matrix for the composites. Hop residue (H) from supercritical carbon dioxide extraction provided by the Łukasiewicz Research Network– New Chemical Syntheses Institute, Puławy, Poland, was used as the filler. The hop residue was ground in a quern grinder, then dried at 105 °C for 4 h, and immediately before weighing run through a 4 mm sieve to separate the agglomerates remaining after heating. The filler content was 10, 20 and 30 wt % in PVC/10H, PVC/20H and PVC/30H composites, respectively.

### 2.2. Sample Preparation

Dry blend PVC and PVC/H composites were processed by twin-screw extrusion using EHP 2 × 24M extruder with L/D = 40 from Zamak Mercator, Skawina, Poland. The temperatures of the following cylinder zones were: 100, 110, 120, 130, 140, 150, 160, 170 and 175 °C, and the head temperature was 170 °C. The extrudates were milled using a Rapid 150 knife mill (Rapid Group, Bredaryd, Sweden). The milled materials were used to produce the dumbbell-shaped specimens 1A type according to the PN-EN ISO 527-1,2 standard, using an ENGEL Victory 120 injection molding machine (ENGEL, Schwartzberg, Austria). The following parameters were used: injection rate 20 mm/s, clamping time 15 s and cooling time 40 s. The temperatures of successive cylinder zones were: 160, 170, 175 and 180 °C and the mold cavity 20 °C. Beams for impact tests were also cut from the dumbbell-shaped specimens according to PN-EN ISO 179-1.

### 2.3. Testing Methods

#### 2.3.1. Morphology of PVC Composites

Observations of the macrostructure of PVC and the composites were carried out using scanning electron microscopy (Hitachi TM 3000 SEM, Hitachi Ltd., Tokyo, Japan). Fracture profiles were obtained after cooling in liquid nitrogen and an impact break. Before the observations, the samples were dusted with a layer of gold with palladium. The observations were conducted using a voltage of 5 keV.

#### 2.3.2. Chemical Structure

FTIR Nicolet 6700 spectrophotometer (Thermo Electron Corporation, Waltham, MA, USA) with the ATR attachment (suppressed total reflection) was used to describe the chemical structure of the filler and composite samples. Absorption spectra of these materials were obtained by scanning each sample 64 times in the range of wave number 4000–400 cm^–1^. The OMNIC 8.2.0.387 program from Thermo Fisher Scientific Inc. (Waltham, MA, USA) was used to analyze the FTIR spectra.

#### 2.3.3. Thermal Analysis of PVC Composites

Differential scanning calorimetry (DSC) thermal analysis of samples of PVC and its composites with hop residue was performed with the DSC Q1000 of TA Instrument (New Castle, DE, USA). Samples of approximately 6 mg were placed in aluminum crucibles and heated in a helium atmosphere at a rate of 10 °C /min over a temperature range of 20 to 100 °C. The temperature accuracy of the device was 0.1 °C. The glass transition value was estimated at the DSC base line inflection point as the midpoint of the tangent to the curve.

Two types of analyses were performed to evaluate the thermal stability of the materials. Static thermal stability tests of PVC and PVC/H composites using Congo red were carried out at 180 °C in accordance with the ISO 182-1:1990 standard.

In addition, thermogravimetric analysis (TGA) of the filler, PVC and its composites was performed. Samples of 10 ± 1 mg were tested under a nitrogen atmosphere using the TGA Q500 (TA Instrument, New Castle, DE, USA). Investigations of the filler were carried out at the rate of 10 °C/min from room temperature to 950 °C; for PVC and PVC/H composites, from room temperature to 600 °C. The mass accuracy and temperature precision of the device were 0.5% and 0.1 °C, respectively. The Universal Analysis 2000 version 4.7A application from TA Instruments was used to analyze the results from the TGA tests.

#### 2.3.4. Density Determination

The density of PVC composites with hop residue was determined using the Pycnomatic helium pycnometer from Thermo Fisher Scientific (Waltham, MA, USA) according to PN-EN ISO 1183-3. Five specimens from each composite type were tested.

#### 2.3.5. Mechanical Properties

Tensile strength was studied according to ISO 527-1 standard using Instron 5967 testing machine (Instron, Norwood, MS, USA) equipped with a video extensometer. The samples were tensioned at the 1 mm/min to 0.25% strain and then at 50 mm/min. The results were the arithmetic means of 10 tests for each type of composite.

The bending tests for 10 specimens of each composite type were performed according to PN−EN ISO 178 using the same tensile machine equipped with a three-point bending rig. The vertical displacement speed of the rig during the test was 2 mm/min to 0.25% strain and then 10 mm/min. The span was 64 mm.

The Charpy impact resistance was determined according to PN-EN ISO 179-1 with a CEAST 9050 Impact Pendulum (Instron, Norwood, MS, USA) using a hammer impact energy of 1 J. Unnotched bars: 80 mm in length, 10 mm in width and 4 mm in height were applied. The results were the arithmetic means of 10 tests for each type of composite.

The Rockwell hardness was tested with a ZWICK 3106 hardness tester (ZWICK GmbH & Co. KG, Ulm, Germany), in accordance with EN 10109-1 standard. The indenter load equaled 358 N. The results were the arithmetic means of 10 tests for each type of composite.

#### 2.3.6. Flammability of PVC Composites

The fire behavior of the PVC composites was characterized using a mass loss calorimeter (MLC) from FTT Ltd. (East Grinstead, UK) according to ISO 13927. The samples with the dimensions of 100 × 100 × 4 mm were tested by applying a heat flux of 50 kW/m^2^ and the distance from the ignition source of 25 mm.

The limiting oxygen index (LOI) for the 10 samples of each type of composite was determined according to the standard EN ISO 4589-1 using an instrument of Fire Testing Technology Ltd. (East Grinstead, UK). Samples in the form of 100 × 10 × 4 mm bars were used for the study.

The UL94 flame tests were carried out in a chamber produced by FTT Ltd. (East Grinstead, UK). The measurements were determined according to the UL94 test standard with vertical sample beam position and a methane-fed burner of 20 mm height. A flame was applied to the center of the lower edge for 10 s and removed. If burning ceased within 30 s, the flame was reapplied for an additional 10 s.

## 3. Results

### 3.1. Filler Testing Results

**Figure 1:**
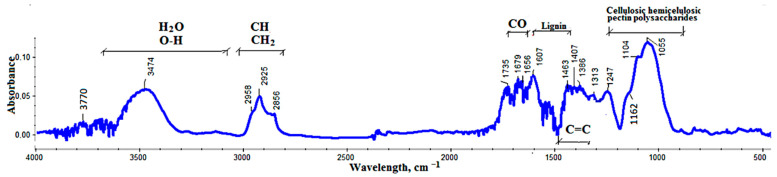
FTIR spectrum of hop residue.

Hop residue after extraction was used to fill the PVC composites and characterized using ATR-FTIR (Figure 1). A description of the characteristic bands in the spectrum of the hop residue is shown in Table 1.

On the FTIR spectra, several ranges of bands from the vibration of groups that are characteristic for plant particles were visible (Figure 1). The first band was associated with vibrations of OH groups in the 3030–3650 cm^–1^ range; the second with vibrations of CH, and CH_2_ groups; the third with vibrations of C=O groups; the fourth with vibrations of C=C groups in aromatic compounds; the fifth in the 900–1200 cm^–1^ range resulting from vibrations of various groups in lignocellulosic materials. To describe the primary components in the hop residue, bands in the 900–1650 cm^–1^ range were analyzed. In the work of Adapa [51,52], bands characteristic of these materials were described on the basis of studies of pure cellulose, hemicellulose and lignin and literature analyses. The results of this analysis were compared with the description of bands characteristic for primary components (cellulose, hemicellulose and lignin) in the spectra of the hop residue (Table 2).

**Figure 2:**
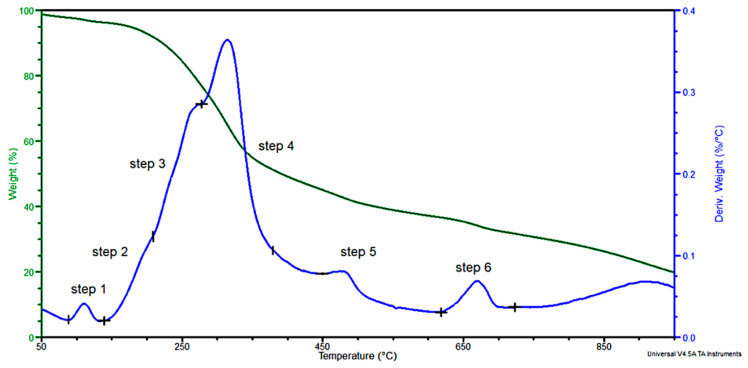
Thermograms of hop residue.

Most of the bands in the spectra of pure cellulose, hemicellulose and lignin were visible in the hop residue FTIR spectra. Thermal degradation was analyzed under an inert atmosphere and the results are shown in Figure 2, while the results from the analysis of mass change (TG) and derived mass change (DTG) curves are shown in Table 3.

TG curves were used to determine the temperature of 5% weight loss (T_5%_), weight loss at 180 °C and the residue after degradation at 750 °C. In turn, the DTG curves show the temperature of the maximum degradation rate at each stages of degradation (T_max1_, T_max2_, T_max3_, T_max4_, T_max5_ and T_max6_), maximum degradation rate at these temperatures (V_max1_, V_max2_, V_max3_, V_max4_, V_max5_ and V_max6_) and the mass change at each stage (Δm_1_, Δm_2_, Δm_3_, Δm_4_, Δm_5_ and Δm_6_), respectively.

In the hop residue about 180 °C, there was a mass loss of about 5% associated with the release of easily volatile substances (mostly water), other easily volatile low-molecular weight compounds, lipids and proteins. These products can affect the extrusion of the composites. After hop residue degradation, about 30 wt % of various forms of carbon may promote the formation of char during combustion. In the filler, 2.2% of the water absorbed by the hop residue and 1.4% of the water bound by hydrogen bonds (first degradation step) were lost through dehydration [53,54]. This step is associated with the vaporization of volatile components and non-polymeric constituents, i.e., lipids and proteins [55], and during this stage, about 5 wt % was lost. The next degradation stage occurred from 215 to 280 °C, corresponding to a weight loss of 15%. Most of the degradation products formed in this stage probably came from the degradation of hemicellulose. The next degradation stage was completed at about 370 °C, during which about 29.5 wt % of the hop residue was lost, and most of the degradation products came from the pyrolysis of cellulose [53]. However, it is important to note that for lignocellulosic components, a range between 220 and 380 °C occurred in the decomposition of cell-wall biopolymers: pectin, hemicellulose, cellulose and lignin [56,57]. In the next—fifth—stage of degradation, there was a mass loss of about 8.5%. The degradation rate in this step was four times lower than in the fourth, probably due to a lower (1319 kcal/mol) activation energy of thermal lignin degradation compared to that of cellulose [57]. In the 380–620 °C range, the decomposition of lignin was observed although it could start as early as about 160 °C and last up to 900 °C [58]. However, the highest rate of lignin degradation was achieved in the 380–460 °C range [59]. The next degradation stage was completed at about 725 °C. This step can be attributed to the decomposition of inorganic compounds and the dehydrogenation and aromatization of char [60,61,62]. Approximately 5 wt % was lost.

**Figure 3:**
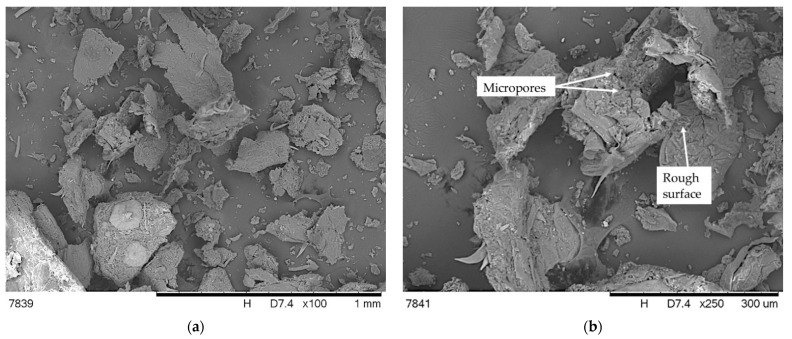
SEM images with different magnification of hop residue (**a**,**b**).

Figure 3 shows SEM images of hop residue after grinding and drying at different magnifications.

SEM images of hop residue show variation in the shape and size of their particles (Figure 3a). Perpendicular shaped particles are visible, but also elongated fiber particles. This indicates that they may be the fragments of leaves, stems and, in the case of hop, cones. An analysis of the photograph at a higher magnification indicated that the particles had sizes ranging from several to 250 µm, and micropores were visible inside (Figure 3b). Moreover, it was found that the filler surface was rough with numerous irregularities, which can positively influence the mechanical adhesion between polymer and filler.

### 3.2. Results of Composite Tests

**Figure 4:**
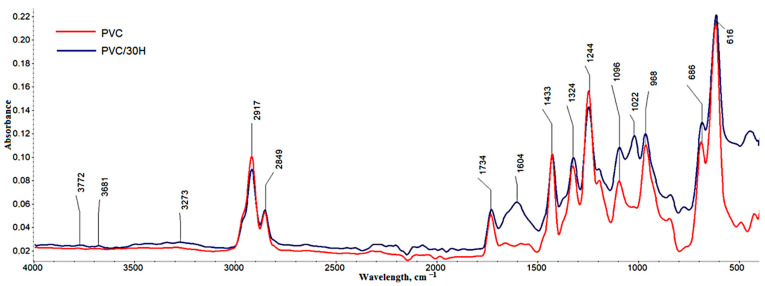
FTIR spectra for PVC and its composites with 30% wt. of residual hops.

Figure 4 shows the FTIR spectra of poly(vinyl chloride) and its composites with description bands characteristic of PVC and those visible in the spectra of the composites from the introduced hope residues.

Figure 4 shows the PVC spectrum with characteristic bands in the 2849–2961 cm^–1^ range derived from C–H stretching vibrations in CH–Cl and CH_2_ groups. The bands at 1433, 1324 cm^–1^ corresponded to CH_2_ deformation wagging, while 1244 cm^–1^ corresponded to CH deformation rocking. On the other hand, the band at 1096 cm^−1^ was associated with the C–C stretch bond on the PVC backbone chain. Other characteristic bands were 968 cm^–1^, responsible for C–H wagging vibrations, and 830, 686 and 616 cm^–1^ derived from C–Cl stretching [17,46]. There was also a distinct band at 1734 cm^–1^ associated with the acrylic additives used in the PVC mixture.

The spectrum of the PVC composite containing 30% hop residue showed a band related to the presence of water introduced with the filler and intermolecular-bonded O–H stretching vibrations centered at 3273 cm^–1^ (Figure 4). There were also new characteristic bands associated with the presence of lignocellulosic filler. The band at 1022 cm^–1^ originated from bond vibrations of C–O groups in cellulose, hemicellulose and methoxyl groups in lignin [44,51,52]. There was also a clear increase in the intensity of the band at 1096 cm^–1^ derived from C–O groups with aromatic backbone vibrations in lignin. In the 1500–1700 cm^–1^ range, a 1604 cm^–1^ band appeared derived from C=C and C=O groups associated with the aromatic lignin rings (Table 1) [51,52].

**Figure 5:**
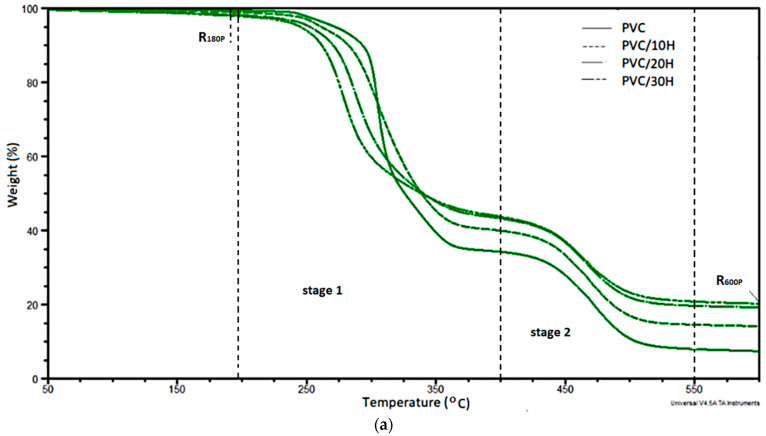

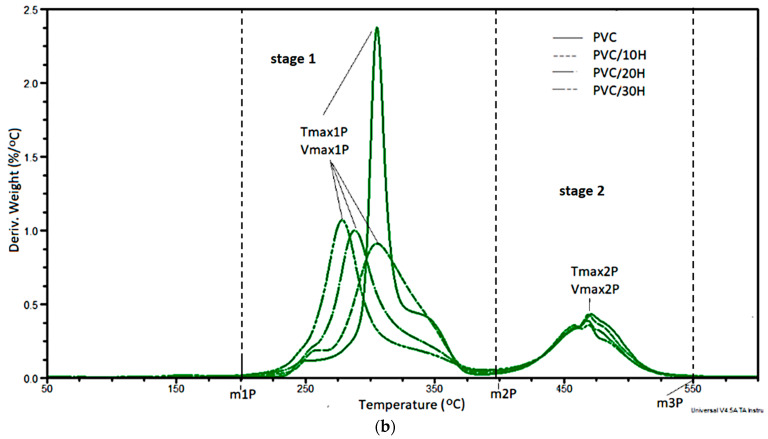
Thermograms of unfilled PVC and PVC composites: (**a**) TG and (**b**) DTG.

A major problem in PVC processing is the significant thermal instability under melt processing conditions. A knowledge of PVC thermostability is particularly important for production using conventional techniques such as extrusion or injection molding. The addition of filler, including those of natural origin, may influence the thermal stability of PVC matrix blends. Therefore, in this study, the thermal stability of the produced materials was determined by Congo red and TGA. Based on a Congo red study of composites with hops, it was found that after the introduction of the filler, the thermal stability increased from 22 min for unfilled material to 100 min for composites with the highest filler content. Such a significant improvement of thermal stability after filler introduction is an extremely advantageous feature of these materials. The probable reason for the longer stability time was that the introduced material had a much lower thermal conductivity. The improvement of thermal stability may be related to the reaction between hydrogen chloride emitted from PVC chains and methyl groups from the methoxyl groups bound to the lignin phenolic rings [63,64]. A similar effect was found for the composites of rigid PVC with lignin treated with the copolyacrylate [65]. The authors concluded that the improved thermal stability of PVC was related to the sterically hindered phenol structure of lignin. The influence of hop residue on the thermal stability of PVC was also characterized based on the TGA thermograms (Figure 5a,b). Table 4 summarizes the results of the analysis of curves of mass change (TG) and the derivative of mass change (DTG).

On the basis of the TG curves, the temperature of 2% mass loss (T_2%P_), the mass loss at 180 °C (R_180P_) and the degradation residue at 600 °C (R_600P_) were determined. In turn, the DTG curves were used to determine the temperature of the maximum degradation rate at each stage (T_max1P_, T_max2P_), the maximum degradation rate at temperatures (V_max1P_, V_max2P_) and the mass change at each stage: Δm_1P_ = m_1P_–m_2P_, Δm_2P_ = m_2P_–m_3P_, respectively. The results of the analysis of the changes in these parameters are summarized in Table 4.

In PVC processing, the temperature range associated with initial degradation is important; therefore, the temperature at which the 2% weight loss of the composites occurred was determined. The temperature of 2% weight loss decreased significantly with increasing the filler content in the PVC matrix (for a composite containing 30 wt % of filler T_2%P_ decreased by 89 °C), which was related to the decomposition of easily volatile substances contained in the hop residue, including water. This is, however, of minor importance since the T2%P values for composites containing 10 wt % and 20 wt % were, respectively, 40 °C and 10 °C higher than the temperature at which PVC is routinely processed that is below 200 °C. Due to the processing temperature parameters used in this study (maximum temperature value during injection molding 180 °C), the weight loss at 180 °C was also determined. It should be stressed that the weight loss at 180 °C was only 1.7 wt % for the composite with the highest filler content, i.e., 30 wt %. The degradation of PVC and the composites occurred in two stages [66,67,68]. During the first stage (200–400 °C), weight loss on the level of 54–65 wt % was associated with PVC dehydrochlorination and the degradation of organic additives in the PVC blend and filler components: mainly hemicellulose, cellulose and lignin [53,56,57,58,69]. Moreover, the weight loss decreased as the filler content in the matrix increased, which indicated the degradation of the matrix, especially the HCl release reaction, which dominated at this stage. For the composite containing 10% of the filler, the temperature at which the degradation rate reached its maximum value was identical to that of the unfilled PVC; an increase in the filler concentration caused a decrease in its value by 20–30 °C. The decrease in T_max1P_ temperature was likely due to the degradation of part of the hemicellulose and cellulose. At the same time, the introduction of hop residue into the PVC caused a more than two-fold decrease in the composite degradation rate in the first stage. This was probably the result of a hindered heat transfer caused by the introduction of larger amounts of filler.

In the second stage of PVC degradation at temperature 400–550 °C, cyclization and crosslinking reactions of conjugated polyene sequences occurred, leading to the formation of aromatic fractions [70]. In addition, the further degradation of hop residue components, especially lignin, occurred in this temperature range [58]. The degradation rate of PVC/H composites was slightly lower than that of PVC, and such as the temperature at which the rate was maximum, hardly at all depended on the filler concentration in the matrix. However, in each case, these values were slightly lower than unfilled PVC. In addition, the TGA results indicated that the residue at 600 °C increased significantly with hop residue loading and was almost three times higher for the composite with 30% hop residue compared to the char yield of unmodified PVC.

**Figure 6:**
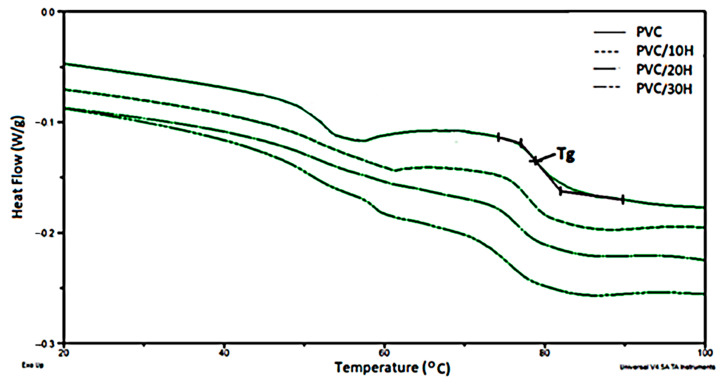
DSC thermograms of PVC and PVC composites.

To evaluate the filler effect on the glass transition temperature (T_g_), a thermal analysis of the PVC samples and PVC/H composites was carried out by the DSC method. The value of Tg determined from DSC thermograms (Figure 6) decreased with an increasing filler concentration from 79 °C for unmodified polymer to 75.6 °C for the composite with a 30% hop content (Table 5). A slight decrease in the temperature range of the glass transition of PVC may indicate a slight plasticizing effect of the residues of lipids, alpha and beta acids, fatty acids and others, introduced into the matrix with the filler.

An endotherm observed on thermograms in the 45–65 °C range was related to the melting of one of the PVC blend components described in [17].

**Figure 7:**
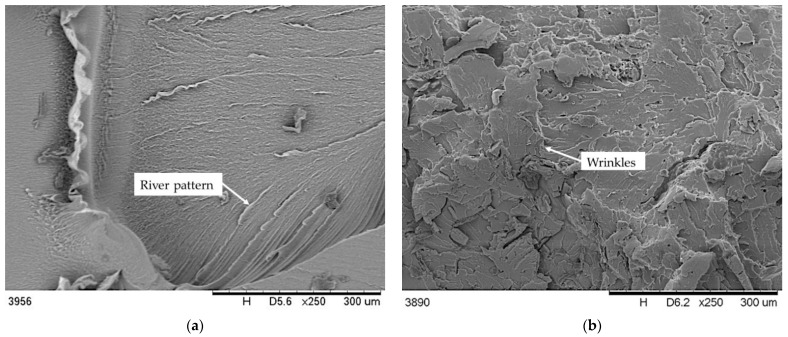

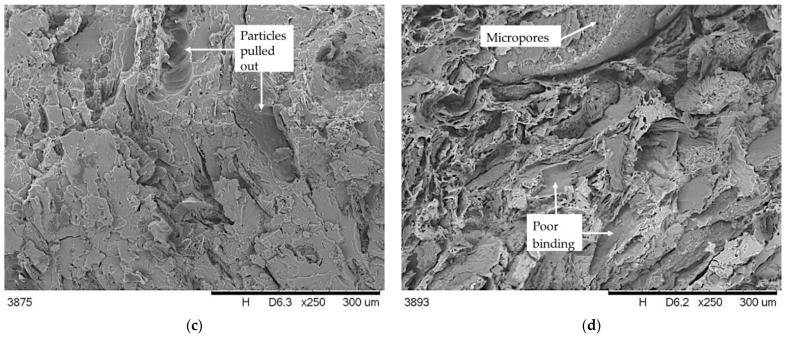
SEM microphotographs of PVC (**a**) and PVC containing hop residue: (**b**) PVC/10 H, (**c**) PVC/20 H, (**d**) PVC/30 H.

Figure 7 shows selected SEM images of the unmodified PVC and PVC/H composites. The layered character of the fracture surface shown in Figure 7a is typical for the processed rigid PVC. A smooth surface, free from inclusions of residual granular elements attested to the properly chosen temperature and the shear parameters during gelation [71]. The addition of filler to the PVC matrix caused a clear change in the morphology of the fracture, on the surface of which numerous grooves and striations were visible. It was also found from the SEM images of PVC/H composites that as the filler content in the matrix increased, the surface became rougher and more porous. This nature of the brittle fracture may have been related to the porous structure of the filler (Figure 3b) and the possible release of residual moisture and volatile compounds in the hop residue. What is important is that a uniform distribution of filler particles in the matrix was observed without any visible agglomerates, which also confirmed the proper processing procedure for obtaining a homogeneous structure of the composites. Moreover, the processing by the extrusion method resulted in the reduction in the maximum filler particle size to about 150 µm, similar to that of other PVC composites, with the addition of natural fillers [72,73]. SEM photographs of the PVC/hop composites disclosed the presence of smooth cavities formed after pulling out the filler, in addition to gaps and voids around it, which indicated a weak interaction between the matrix and filler and may have contributed to the stress concentration in these areas, which would explain the reduction in some of the strength properties of the composites (see section below) [74,75].

**Figure 8:**
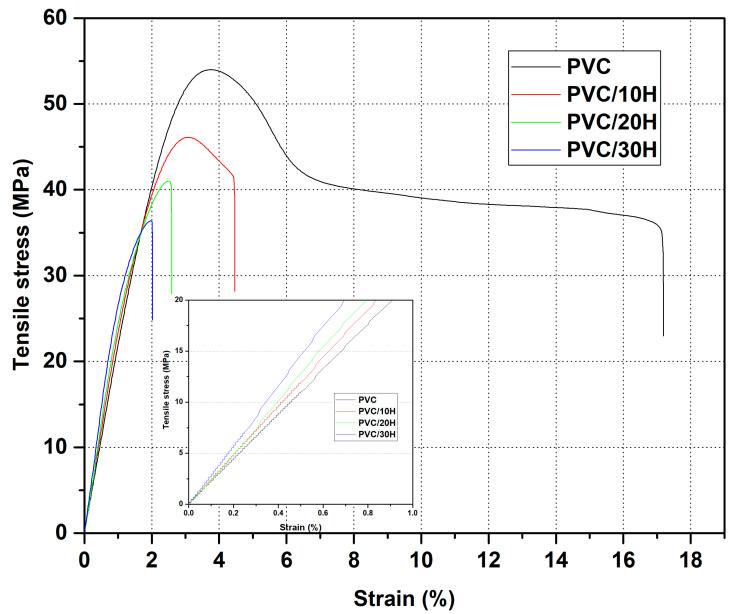
Representative stress–strain curves from the tensile test for the PVC composites.

Table 6 shows the results of density (D) measurements of PVC and PVC/H composites and the strength properties determined during static tensile testing. Based on the test, the Young’s modulus (E_t_), stress at yield (σ_y_), strain at yield (ε_y_), stress at break( σ_b_) and strain at break (ε_b_) were determined. Figure 8 shows a representative curve for each replicated test.

**Figure 9:**
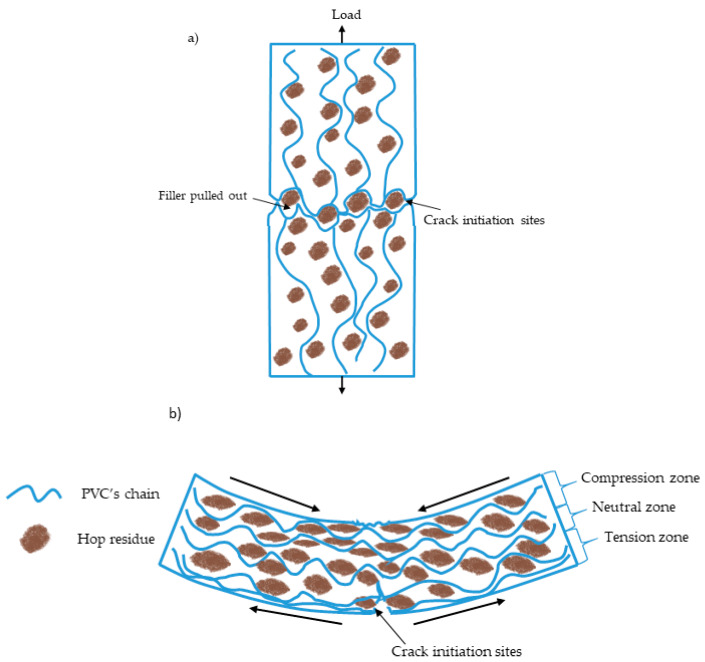
An illustration of the possible fracture mechanism of PVC/H composites in tension (**a**) and bending (**b**).

On the basis of the density test results, it was found that adding hop residue caused a slight increase in the density of the PVC composites. Its value increased only by 0.021 g/cm^3^ for PVC/30H composite in comparison with the unfilled polymer. This small increase in composite density in spite of the porous structure of the filler found on SEM images (Figure 3b and Figure 7b–d) may have been caused by the filling of some pores by molten polymer during the injection molding. Similarly to other PVC composites with natural filler [72,73], the addition of hop residue to PVC caused a decrease in tensile strength such that the higher the filler concentration in the matrix, the lower the value (Table 6). This deterioration of strength can be attributed to the incompatibility of the filler with the matrix, causing the formation of voids and gaps around the filler as observed on the SEM images of brittle fractures (Figure 7b–d) which acted as sites of crack formation and stress concentration (Figure 9a). Moreover, the tensile curves showed that the addition of hop residue clearly changed the stress–strain relationship (Figure 8). In the case of PVC, a clear yield point was seen at about 56 MPa, followed by a decrease in stress and a further increase in strain, resulting in the ductile fracture of the sample. On the other hand, for the PVC/10H composite, no characteristic plateau was observed after the yield point, which indicated an increase in brittleness. In the composites with 20 and 30 wt % hop residue, however, no yield point was observed, and the curve acquired a shape typical of composites with brittleness failure when maximum stress was reached. This was accompanied by a significant decrease in the strain at break from 18.8 for PVC to 4.7, 2.6 and 2.2% for PVC containing 10, 20 and 30 wt % hop residue, respectively. At the same time, Young’s modulus increased by 70.8, 75.4 and 79.0% for PVC/10H, PVC/20H and PVC/30H, respectively, indicating a significant increase in the stiffness of the composites. Similar relationships were observed by Abdellah et al. [75], who studied PVC–wood flour–calcium carbonate composites.

Table 7 shows the results of the Rockwell hardness (HR) and Charpy impact strength (KC) as well as the strength properties during the static bending test. Based on the tests, the following were determined: bending stress (σ_f_), bending strain (ε_f_) and elastic modulus (E_f_).

**Figure 10:**
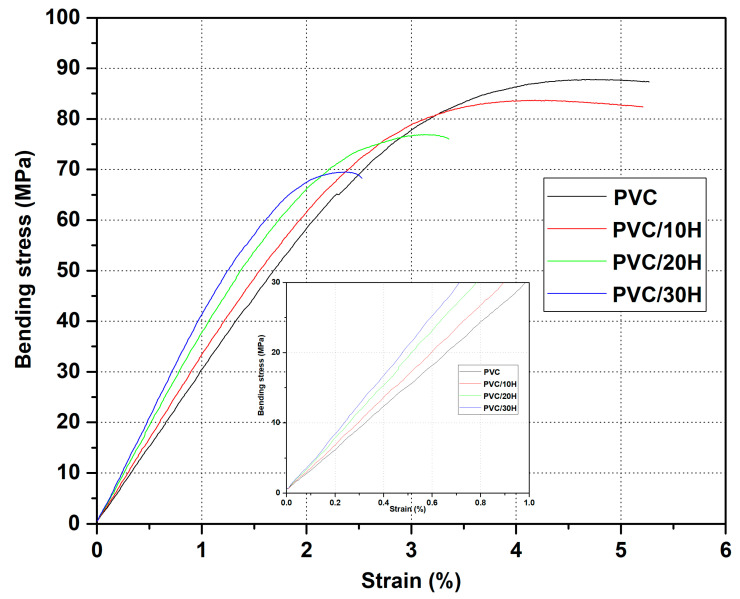
Representative stress–strain curves from the bending test for the PVC composites.

Similar to the tensile strength, the addition of hop residue caused a decrease in flexural strength. However, the recorded changes were much smaller than for the maximum tensile stress, as the maximum flexural stress decreased by 1.7, 8.8 and 17.3% for PVC/10H, PVC/20H and PVC/30H, respectively. This may be related to the fact that three-point bending causes compression and tension in the material (Figure 9b). Literature data indicate that WPC composites have an increased compressive strength than unfilled thermoplastics, even in the case of poor adhesion between filler and polymer [76,77]. As a result, the flexural strength of composites containing up to 20 wt % H was at the level of the reference sample. Increasing the H content to 30 wt % resulted in a marked decrease in flexural strength due to the significant limitation of the matrix to deformation. On the other hand, as expected, the addition of hop residue caused an increase in the modulus of elasticity, which changed linearly with an increasing filler content. It was also confirmed by the stress–strain curves (Figure 10). In the case of the PVC/10H composite, the curve was similar to that of the unmodified PVC. As a result for these two materials, the measurement was carried out until a deflection arrow of 6 mm was obtained since no fracture of the specimen had occurred. In contrast, the addition of 20 and 30% hops caused the slope of the curve in the proportionality region to become greater, indicating an increase in stiffness, which was characterized by a brittleness failure before the arrow was obtained. This behavior of the materials also affected the strain values, which decreased by 17.1, 39.5 and 55% compared to the unfilled PVC. According to literature data, the increase in stiffness is accompanied by an increase in hardness [78]. As seen in Table 7, the addition of 10% hop residue did not change this value, while for PVC/20H and PVC/30H, there was an increase of 8.8 and 12.8%, respectively. The results confirmed that the addition of hop residue caused an increase in composite stiffness, which could be attributed to the reduced mobility of the polymer chains. However, the increase in the modulus of elasticity was less pronounced than Young’s modulus, which again can be explained by the bending behavior of the specimen, as this test combined compression and tension of the specimen. Furthermore, it has been found that natural fillers can deteriorate compressive stiffness [76]. The reduction in chain flexibility can be explained by a reduction in impact strength, which agrees with the literature data [79,80,81].

**Figure 11:**
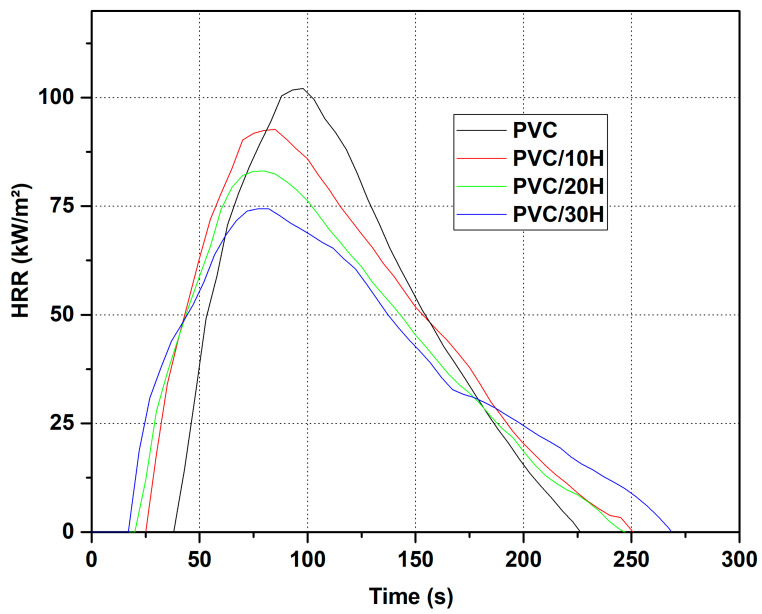
HRR vs. time for PVC and PVC composites.

Poly(vinyl chloride) is frequently used in construction, and its suitability is often determined by its relatively high fire resistance [82]. Its modification process often leads to a lower LOI [22]. Therefore, the fire resistance of the fabricated composites was evaluated, and the results are summarized in Table 8 and Figure 11.

The value of the oxygen index for the unmodified PVC was 37.4%. The introduction of hop residue into this polymer reduced the LOI to 32.3% for a composite containing 30 wt % filler. The LOI values were due to the organic nature of the filler and correlated with the results presented by other researchers. Furthermore, as with flexural and tensile strengths, the reduction in LOI values may be due to the porous structure of the composite and filler which promotes the migration of oxygen and flame into the material. It is worth noting that, despite the reduction in LOI, all composites were characterized by flammability class V0, which still made them attractive for fire resistance. Flammability tests were also performed using a cone microcalorimeter. During the analysis, the characteristic parameters such as tti (time to ignition), pHRR (Peak Heat Release Rate), ttpHRR (time to Peak Heat Release Rate), EHC (Effective Heat of Combustion), THR (Total Heat Released) and PML (Percentage Mass Loss) were determined.

The results summarized in Table 8 represent the arithmetic mean of the three measurements for each sample, while the graph shows the representative curves for each sample. The table shows that the hop content increased and the time to ignition of the sample decreased. As a result, the PVC/30H composite had a 44% lower tti than that of the unmodified PVC. One of the reasons was a significant decrease in temperature at the onset of the thermal degradation after the filler was introduced, which was observed during the thermogravimetric analysis. In addition, natural additives reduced the thermal conductivity which led to a rapid temperature rise at the sample surface and a faster ignition [37]. This high temperature behavior of the composites correlates with the LOI results and, again, may be due to a poor compatibility between the filler and polymer and the porous structure. The analysis of pHRR indicated that the addition of hop residue reduced its value. Unmodified PVC was characterized by a pHRR of 105.7 kW/m^2^, while the addition of 10, 20 and 30 wt % hop residue caused a 20.7, 28.0 and 34.4% decrease, respectively. The results were consistent with the literature data for thermoplastic composites with natural filler [35]. On the other hand, EHC and THR did not change significantly for composites containing up to 20 wt % filler. However, increasing the content to 30 wt % resulted in an increase of 33.9 and 22.2%, respectively, compared to unmodified PVC. According to literature data, the EHC refers to the gas-phase combustion of the volatile gases released from the polymer, while PML refers to the carbonization effect in the condensed phase [83]. These effects can be quantified by reducing EHC and PML, respectively. The increased char yield decreased the release of flammable volatiles; in turn, a reduction in EHC indicates flame inhibition or fuel dilution. The EHC of PVC/30H composite increased to 124.4% compared to the unmodified sample. The increase in residue (15.1% instead of 8.0%) slightly reduced the amount of released fuel to 92.3%. Therefore, increasing the THR value to 114.7% after adding 30 wt % of hop residue was related to the reduction in the chlorine content in the mass; as a result, its activity in the gas phase was limited, which was confirmed by the calculated value (124.4 × 92.3 = 114.8). Therefore, the reduction in pHRR was probably related to the protective barrier formed by the hop residue, since the addition of hop residue caused a reduction in PML which coincided with the results of the TGA and char yield. The relative protection effect was calculated to be 38.1% (124.4% × 92.3% × 61.9% = 65.6%) which compared to the charring effect (7.7%) and flame inhibition effect (−24.4%) confirmed that the barrier effect plays a major role in decreasing the pHRR. Similar dependencies were observed in the case of the remaining samples containing 10 and 20 wt % of hope residue. Probably, this ability of the composites to form a char caused them to obtain the V0 flammability class, despite their lower oxygen index and shorter time to ignition.

Based on an analysis using a cone calorimeter, it was possible to calculate the fire growth rate index (FIGRA), the maximum ratio of the HRR to the time it was achieved. The smaller the FIGRA index, the more slowly the fire spreads, which increases the time to evacuate and start extinguishing operations. The FIGRA values confirmed that the addition of hop residues did not significantly affect the rate of fire spread. However, a non-significant decrease in FIGRA was observed with increasing amounts of hop residue. The FIGRA for the tested materials was in the range of 0.96–1.10 (Table 8), while for the reference sample it was 1.10. Such FIGRA and pHRR values in combination with the flammability class V0 indicate that the obtained materials still have a good flame resistance.

## 4. Conclusions

**Figure 12:**
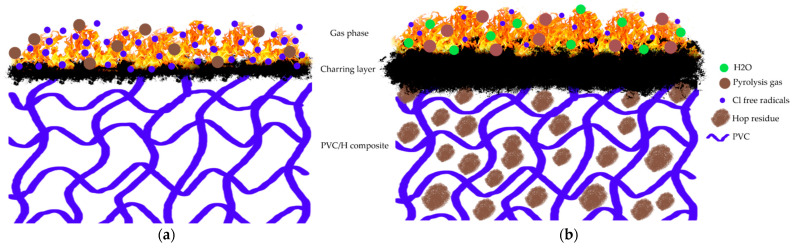
An illustration of the possible mode of flame retardant action in PVC (**a**) and PVC/H composites (**b**).

Currently, poly(vinyl chloride) is, along with polypropylene and polyethylene, one of the most widely produced plastics; therefore, its disposal and recycling are a very important issue. One of the methods of improving the PVC–environment relationship is reducing the use of petrochemical raw materials in favor of natural resources. The use of natural fillers, including agro-waste, for the production of PVC composites is undoubtedly part of this trend. Due to the diversity of individual wastes from food processing, differing in the content of cellulose, lignin, hemicellulose and other secondary components, it is important to accurately determine the impact of this type of filler on the functional properties of composites with a PVC matrix. Therefore, this work analyzed the effect of adding residual hops on the mechanical properties, chemical and physical structure, and the flammability of composites within a rigid poly(vinyl chloride) matrix. Residual hop pellets obtained after supercritical extraction in CO_2_ were used. This residue was characterized by a porous structure (SEM analysis), about 5 wt % moisture or highly volatile lipids and proteins, about 30 wt % cellulose, about 15 wt % hemicellulose and 13 wt % lignin (TGA). This filler was added to PVC in the amounts of 10, 20 and 30 wt %. Based on the results, the composites could be processed by traditional processing (extrusion and injection molding) because the addition of residual hops increased their thermostability, as shown by a Congo red test. Additionally, the thermogravimetric analysis of the composites showed a positive effect of residual hops on the degradation processes during heating, as the degradation rate in the first and second stages decreased compared to the unmodified PVC. This thermal degradation of PVC composites resulted in an increased char yield during thermolysis. The PVC/30H scaling yield was almost three times higher than of pristine PVC. This also translated into the results of the cone microcalorimeter flammability test. The mass loss recorded during the cone microcalorimeter test was lower for the composites which confirms that the addition of H promoted the formation of a stable solid residue constituting a physical protective barrier. Furthermore, the quantitative assessment indicated that the action in the gas phase was limited (EHC increased due to the decrease in chlorine content) and an additional barrier effect played a major role in decreasing the pHRR value (Figure 12). Additionally, the FIGRA values (in the range 1.01–0.96 instead of 1.10), which determine the flame spread, indicated that PVC/H composites have a lower fire risk. In turn, the oxygen index of the composites decreased with increasing amounts of filler, but this did not change the V0 flammability rating. Based on an analysis of mechanical properties, RH addition caused a significant increase in composite stiffness. On the other hand, the tensile and bending strength decreased with the increase in H content, which may result from the poor interaction of the matrix with the filler visible in the SEM photos. It seems that a good way to obtain a good strength is the use of coupling agents or the production of hybrid composites containing, besides fillers, also other nanofillers. Such a connection should allow the complete use of hop residue as a cheap, ecological filler in PVC composites for construction. At the same time, the addition of residual hops helped to offset the environmental damage caused by PVC manufacturing. Manufacturing of PVC/H composites is an alternative solution for the utilization of hop waste after beer production as well as technological and post-use PVC waste. The composites obtained were characterized by mechanical properties sufficient to use them as a material for the production of structural elements.

## Figures and Tables

**Table 1:** FTIR analysis results of hop residue.

Functional Groups	Vibrational Modes	Wave Number, cm^–1^	Assigned Species	Ref.
O–H	Stretch	3770	Phenols, alcohols or carboxylic acids	[44,45]
O–H	Stretch	3474	H_2_O	[44,45,46,47]
CH, CH_2_	Stretch	2925	Long-chain fatty acids, waxes, carotenoids and phytosterols	[44,47,48,49]
CH, CH_2_	Stretch	2856		
C=O	Stretch	1600–1800	Carboxylic acid/ester or aldehyde/ketone groups	[44,49,50]
C=O	Stretch	1735	Carboxylic acids of the ester	[44,49,50]
C=O	Stretch	1607,	The aromatic ring	[50,51]
C=C	Stretch	1350–1600	The aromatic structures	[50,51]
N–H	Bending	1536	Amides and amines group	[50]
C–O	Stretch	1516	Aromatic lignin	[50,51,52]
C–H	Stretch	1463	methyl and methylene group	[50,51,52]
C–O	Stretch	1432	The aromatic structures	[49,51,52]
C–O	Stretch	1386	The aromatic structures	[50,51,52]
C–O	Stretch	1333	The aromatic structures	[50,51,52]
C–H	Wagging	1313	Cellulose	[51,52]
C–O	Stretch	1247	Hemicellulose	[44,51,52]
C−O	Stretch	1203	Hemicellulose	[51,52]
C–O–C	Stretch	1162	Glycosidic linkage	[44,49,51,52]
C–O		1104	Aromatic ring skeleton of lignin	[49,51,52]
C–O–CC−O and/or C−C	Stretch	1055	Phenyl/methyl ether	[49,51,52]

**Table 2:** Characteristic peaks of pure cellulose, hemicellulose, lignin and cellulose, hemicellulose and lignin in hop (H) residue.

Wave Numbers, cm^–1^	Cellulose 100%	H	Hemicellulose 100%	H	Lignin 100%	H
1650–1600	-	-	1606	1607	-	-
1600–1550	-	-	-	-	1599	1607
1550–1500	-	-	-	-	1511	1513
1500–1450	-	-	1461	1463	1467	1468
1450–1400	1431	1432	-	-	1429	1432
1400–1350	1373	1386	-	-	-	-
1350–1300	1319 1338	1313 -	-	-	-	-
1300–1250	-	-	1251	1247	-	-
1250–1200	1203	-	1213	-	-	-
1200–1150	-	-	1166	1162	1157	-
1150–1100	-	-		-	1104	1104
1100–1050	-	-	1050	-	1054	1055

**Table 3:** Results of TG and DTG analysis of hop residue.

Parameter	Value
Temperature at 5% weight loss (T_5%_), °C	177
Mass loss at 180 °C, %	5.2
Residue at 750 °C, %	30.8
Stage 1—release of associated water	
Temperature range, °C	90–140
Mass change (ΔΔm_1_), %	1.4
Temperature at V_max1_ (T_max1_), °C	111
Maximum degradation rate (V_max1_), %/°C	0.04
Stage 2—lipid and protein degradation	
Temperature range, °C	140−215
Mass change (Δm_2_), %	5
Temperature at V_max2_ (T_max2_), °C	*
Maximum degradation rate (V_max2_), %/°C	*
Stage 3—hemicellulose degradation	
Temperature range, °C	215−280
Mass change (Δm_3_), %	15.0
Temperature at V_max3_ (T_max3_), °C	263
Maximum degradation rate (V_max3_), %/°C	0.28
Stage 4—cellulose degradation	
Temperature range, °C	280−370
Mass change (Δm_4_), %	29.5
Temperature at V_max4_ (T_max4_), °C	315
Maximum degradation rate (V_max4_), %/°C	0.36
Stage 5—lignin degradation	
Temperature range, °C	370–620
Mass change (Δm_5_), %	13.4
Temperature at V_max5_ (T_max5_), °C	477
Maximum degradation rate (V_max5_), %/°C	0.08
Stage 6—aromatic compounds degradation	
Temperature range, °C	620−725
Mass change (Δm_6_), %	5.0
Temperature at V_max6_ (T_max6_), °C	671
Maximum degradation rate (V_max6_), %/°C	0.07

* value difficult to determine.

**Table 4:** The results of thermostability analysis by Congo and analysis of TG and DTG curves of poly(vinyl chloride) and PVC composites.

Parameter	PVC	PVC/10H	PVC/20H	PVC/30H
Thermostability determined by Congo method, min	22	90	95	100
Temperature of 2% mass loss (T_2%P_), °C	285	240	210	196
Mass loss at 180 °C, R_180P_, %	0.4	0.8	1.5	1.7
Stage 1				
Temperature range, °C	200–400			
Mass change (Δm_1P_), %	65.3	59.2	55.2	54.5
Temperature at V_max1P_ (T_max1P_), °C	305	305	288	270
Maximum degradation rate (V_max1P_), %/°C	2.38	0.91	1.00	1.07
Stage 2				
Temperature range, °C	400–550			
Mass change (Δm_2P_), %	26.2	25.4	23.5	22.9
Temperature at V_max2P_ (T_max2P_), °C	472	469	468	469
Maximum degradation rate (V_max2P_), %/°C	0.43	0.43	0.39	0.36
Residue at 600 °C, R_600P_, %	7.5	14.3	19.3	20.3

**Table 5:** Glass transition temperature of PVC and PVC-based composites.

Sample	T_g_, °C
PVC	79.0
PVC/10H	78.3
PVC/20H	76.1
PVC/30H	75.6

**Table 6:** Results of density and mechanical properties of PVC and PVC composites with addition of hop residue after extraction.

Sample Symbol	D, g/cm^3^	σ_y_, MPa	ε_y_, %	E_t_, MPa	σ_b_, MPa	ε_b_, %
PVC	1.364 ± 0.002	56.3 ± 3.2	3.81 ± 0.32	2212 ± 54	36.9 ± 1.9	18.81 ± 2.1
PVC/10H	1.368 ± 0.001	48.8 ± 2.8	3.21 ± 0.36	3779 ± 68	43.7 ± 1.4	4.73 ± 0.5
PVC/20H	1.378 ± 0.001	42.5 ± 3.6	2.40 ± 0.41	3879 ± 55	40.5 ± 1.1	2.60 ± 0.3
PVC/30H	1.385 ± 0.001	-	-	3959 ± 71	35.8 ± 1.8	2.15 ± 0.3

**Table 7:** Flexural, hardness and impact test results of PVC and PVC/H composites.

Sample Symbol	σ_f_, MPa	ε_f_, %	E_f_, MPa	HR, N/mm^2^	KC, kJ/m^2^
PVC	86.1 ± 2.4	5.54 ± 0.8	3110 ± 64	106.2 ± 1.8	65.2 ± 1.6
PVC/10H	84.6 ± 3.0	4.59 ± 0.9	3240 ± 35	106.2 ± 2.7	18.6 ± 1.0
PVC/20H	78.5 ± 2.7	3.35 ± 0.5	3610 ± 52	114.9 ± 2.8	18.5 ± 0.7
PVC/30H	71.2 ± 2.1	2.49 ± 0.4	3840 ± 48	119.8 ± 2.6	11.3 ± 0.5

**Table 8:** Flammability test results of PVC composites with the addition of hop residues.

Sample Symbol	LOI, %	UL 94	tt_i_, s	pHRR, kW/m^2^	ttpHRR, s	EHC, MJ/kg	THR, MJ/m^2^	PML, %	FIGRA, kW/m^2^
PVC	37.4	V0	39 ± 3	105.7 ± 3.8	102 ± 3	4.1 ± 0.2	13.5 ± 0.8	92.0 ± 0.1	1.10
PVC/10H	34.1	V0	28 ± 4	88.6 ± 4.0	87 ± 4	4.4 ± 0.4	13.5 ± 1.3	89.0 ± 0.3	1.01
PVC/20H	33.0	V0	18 ± 3	80.5 ± 2.6	77 ± 2	4.4 ± 0.5	14.3 ± 1.3	86.3 ± 0.5	1.05
PVC/30H	32.3	V0	15 ± 1	73.3 ± 1.1	76 ± 1	5.1 ± 0.7	16.1 ± 2.1	84.9 ± 0.6	0.96

## Data Availability

Not applicable.
